# Comprehensive Review of *Olea europaea*: A Holistic Exploration into Its Botanical Marvels, Phytochemical Riches, Therapeutic Potentials, and Safety Profile

**DOI:** 10.3390/biom14060722

**Published:** 2024-06-18

**Authors:** Hamza Elhrech, Oumayma Aguerd, Chaimae El Kourchi, Monica Gallo, Daniele Naviglio, Imane Chamkhi, Abdelhakim Bouyahya

**Affiliations:** 1 Laboratory of Human Pathologies Biology, Department of Biology, Faculty of Sciences, Mohammed V University in Rabat, Rabat 10106, Morocco; hamza.elhrech@um5r.ac.ma (H.E.); oumayma.aguerd@um5r.ac.ma (O.A.); 2 Laboratory of Materials, Nanotechnology and Environment, Faculty of Sciences, Mohammed V University in Rabat, Rabat 10106, Morocco; elkourchichaimae@gmail.com; 3 Department of Molecular Medicine and Medical Biotechnology, University of Naples Federico II, Via Pansini 5, 80131 Naples, Italy; 4 Department of Chemical Sciences, University of Naples Federico II, Via Cintia, 4, 80126 Naples, Italy; naviglio@unina.it; 5 Geo-Biodiversity and Natural Patrimony Laboratory (GeoBio), Geophysics, Natural Patrimony, Research Center (GEOPAC), Scientific Institute, Mohammed V University in Rabat, Rabat 10106, Morocco; chamkhi.imane@gmail.com

**Keywords:** *Olea europaea*, pharmacological action, secoiridoids, oxidative stress, toxicology, microbial infection

## Abstract

Human health is now inextricably linked to lifestyle choices, which can either protect or predispose people to serious illnesses. The Mediterranean diet, characterized by the consumption of various medicinal plants and their byproducts, plays a significant role in protecting against ailments such as oxidative stress, cancer, and diabetes. To uncover the secrets of this natural treasure, this review seeks to consolidate diverse data concerning the pharmacology, toxicology, phytochemistry, and botany of *Olea europaea* L. (*O. europaea*). Its aim is to explore the potential therapeutic applications and propose avenues for future research. Through web literature searches (using Google Scholar, PubMed, Web of Science, and Scopus), all information currently available on *O. europaea* was acquired. Worldwide, ethnomedical usage of *O. europaea* has been reported, indicating its effectiveness in treating a range of illnesses. Phytochemical studies have identified a range of compounds, including flavanones, iridoids, secoiridoids, flavonoids, triterpenes, biophenols, benzoic acid derivatives, among others. These components exhibit diverse pharmacological activities both in vitro and in vivo, such as antidiabetic, antibacterial, antifungal, antioxidant, anticancer, and wound-healing properties. *O. europaea* serves as a valuable source of conventional medicine for treating various conditions. The findings from pharmacological and phytochemical investigations presented in this review enhance our understanding of its therapeutic potential and support its potential future use in modern medicine.

## 1. Introduction

In ancient times, humans and plants shared a close relationship that encompassed various uses such as food, medicine, and other applications. The olive tree, or *Olea europaea* L., is an evergreen member of the *Oleaceae* family. The shrub is known for its fruit and is primarily grown in Mediterranean regions [1]. Currently, 98% of olive products originate from the Mediterranean basin, playing a significant role in the region’s economy. Beyond their economic value, olive trees offer nutritional and medicinal benefits [2]. Notably, it is the sole species within its genus utilized as a food source [3]. They are typically found along the coastlines of the Mediterranean basin’s eastern region, southeastern Europe, northern Iran near the Caspian Sea’s southern end, western Asia, and northern Africa [4]. One important aspect of the Mediterranean diet is its abundant consumption of phytonutrients, including natural phenols and vitamins [5]. Scientists are keenly interested in these compounds due to their ability to activate various signaling pathways involved in regulating metabolism, DNA repair, protein balance, antioxidant defenses, and combating numerous diseases [6]. The main products from the olive, such as leaves, fruit, seeds, olive oil, and olive pomace, possess a diverse chemical makeup. They can be classified according to their fundamental molecular qualities, such as lignans, secoiridoids, flavonoids, and simple phenols and acids [7]; these include oleuropein, flavonols such as rutin and catechin, substituted phenols (vanillic acid, tyrosol, vanillin hydroxytyrosol, and caffeic acid), as well as a range of flavones (diosmetin, diosmetin-7-glucoside, luteolin, and apigenin-7-glucoside) [8]. Also, olive leaves contain a variety of chemicals, including related secoiridoids and oleuropein, which is the main chemical [9]. Among these compounds, oleuropein is commonly cited as the predominant one. Flavonoids may also be present in significant quantities [10]. Chemically, olive tree extracts display an array of pharmacological properties; these encompass anti-obesity, antidiabetic, antimicrobial, anti-inflammatory, anticancer, and immunosuppressive effects. These extracts are efficient in combating both Gram-positive and Gram-negative bacteria [11,12], as well as fungal strains [13,14]. Biological investigations have shown that olive extracts effectively inhibit enzymes involved in the glucose metabolism, proving its antidiabetic efficacy [15,16,17] and potent antioxidant effects [18,19]. Moreover, it displays potent cytotoxic activity, reducing the viability of tumor cells [20,21]. Importantly, investigations have found no toxic effects associated with olive extract consumption [22]. Several factors can influence the qualitative and quantitative phenolic content of olive leaves, such as harvesting time, drying conditions, growing location, extraction process, and cultivar. This review aims to summarize the key studies on the rich and diverse chemical composition of *O. europaea* extracts. It will discuss findings from both in vivo and in vitro investigations of its biological activities, including anticancer, antidiabetic, antimicrobial, and antioxidant effects.

## 2. Investigative Methodology

The data collection of the botanical characterization and classification, distribution, phytochemistry, ethnobotany, and pharmacology of *Olea europaea* utilized various scientific search platforms, such as Web of Science, Google Scholar, PubMed, and Scopus. Only English-language articles published between 2010 and 2024 were included, along with some additional information from the 1990s. In this review, the collected data were methodically arranged, categorized, examined, and condensed by each relevant field. A bibliometric analysis was carried out utilizing multiple search terms linked to *Olea europaea*, such as *Olea europaea* vegetable oil, olive leaf essential oils, *Olea europaea* extracts, biological effects of *O. europaea*, in vivo and in vitro studies, chemical composition, and the genus *Olea*. For plant chemistry data, ChemDraw Pro 17.0 software was used to construct chemical structures and the PubChem database was used to confirm the IUPAC designations of the chemical compounds discovered.

## 3. Results and Discussion

### 3.1. Taxonomy, Botany, Morphology, and Ecology Description of Olive

The olive tree, scientifically known as *Olea europaea* L., places it within the kingdom of green plants, with a more specific categorization in the subkingdom Tracheobionta and the superdivision Spermatophyta. It belongs to the division Magnoliopsida and the subclass Asteridae. Its order can be either Scrophulariales or Lamiales, the *Oleaceae* family. The genus is *Olea*, and the species is *europaea* [23]. Morphologically, the olive tree typically reaches heights of up to 10 m. Its stem exhibits a considerable diameter and often has a twisted or bent appearance. The leaves are smooth, tapering, lanceolate, short, ovate, thin, leathery, and oblong. They have a silvery whitish hue. The petiole measures 1 to 3 cm in width and 5 to 10 cm in length. Abundant small hermaphrodite flowers are present, with a milky white hue. The calyx is brief and bears four little teeth, while the corolla measures 1 to 2 mm in length. The fruit is ovoid and tiny, turning blackish-violet when ripe, typically measuring 1 to 2.5 cm in length. Cultivated kinds tend to have larger fruit compared to wild plants, and the bark’s color is often light gray [24]. The olive tree is adaptable to a range of climates. It thrives at an optimal temperature of 40 °C, with growth occurring between 15 °C to 20 °C. However, high-altitude areas are unsuitable due to the risk of frost. Olive cultivation is feasible even in arid and calcareous soils. Nevertheless, sandy loam soils with appropriate levels of phosphorus, nitrogen, and potassium are the most conducive for olive growth. Olive trees are known for their resilience to drought conditions, and they excel in soils with a pH level lower than 8.5. Additionally, these plants can flourish in soils with elevated boron content without experiencing toxicity issues [25].

### 3.2. Geographical Location of Olea europaea L., in the World

**Figure 1:**
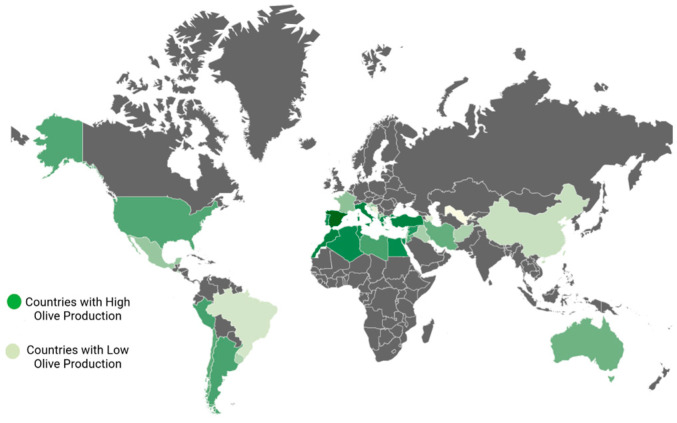
Worldwide geographical locations of olive cultivation.

The Mediterranean climate, characterized by warm, arid summers and cool, wet winters, is one that olive trees can withstand. The olive tree is a thermophilic species, well-suited to endure both drought and salinity challenges [26]. It thrives in a variety of soil types, though it exhibits a preference for sandy loam soils that have a moderate depth [3]. Olives are cultivated in the following regions across the globe (Figure 1): in Asia, you can find olive cultivation (in countries such as China, India, Iran, Syria, Pakistan, Jordan, Palestine, Israel, Iraq, Lebanon, and Turkey); in Oceania, olive trees are grown (in New Zealand and Australia); in the Americas, olives thrive (in the United States, Mexico, Uruguay, Chile, Peru, and Argentina); in Europe, olive cultivation is prominent (in Greece, Italy, Spain, Cyprus, Montenegro, France, Portugal, Croatia, and Albania); Lastly, in Africa, you will find olive trees flourishing (in Morocco, South Africa, Tunisia, Egypt, Libya and Algeria) [27,28].

### 3.3. Worldwide Naming of Olive Varieties

The cultivated *O. europaea* worldwide exhibits various distinct varieties, and the nomenclature of these varieties often evolves from one country to another, reflecting traditional specifications and local practices, as presented in Table 1.

### 3.4. Phytochemistry Characteristics

**Figure 2:**
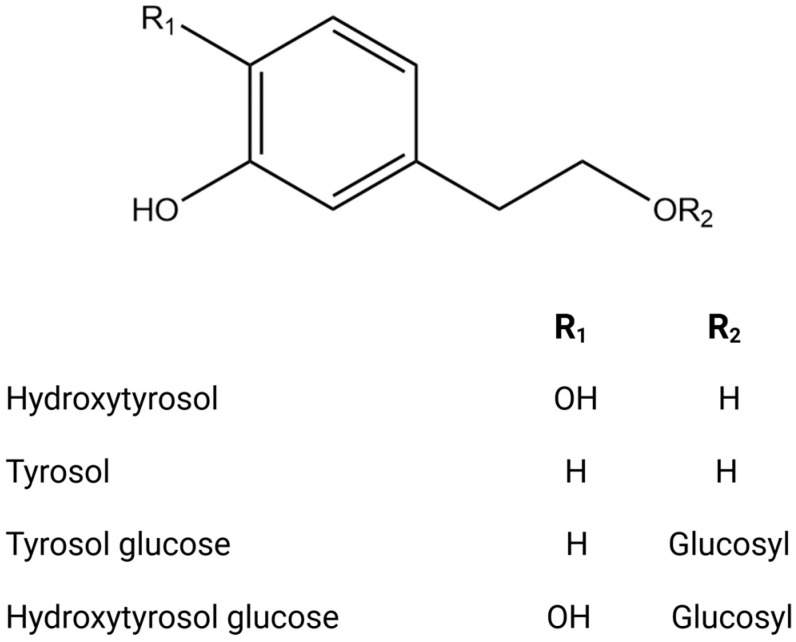
Chemical structures of key phenolic alcohols.

**Figure 3:**
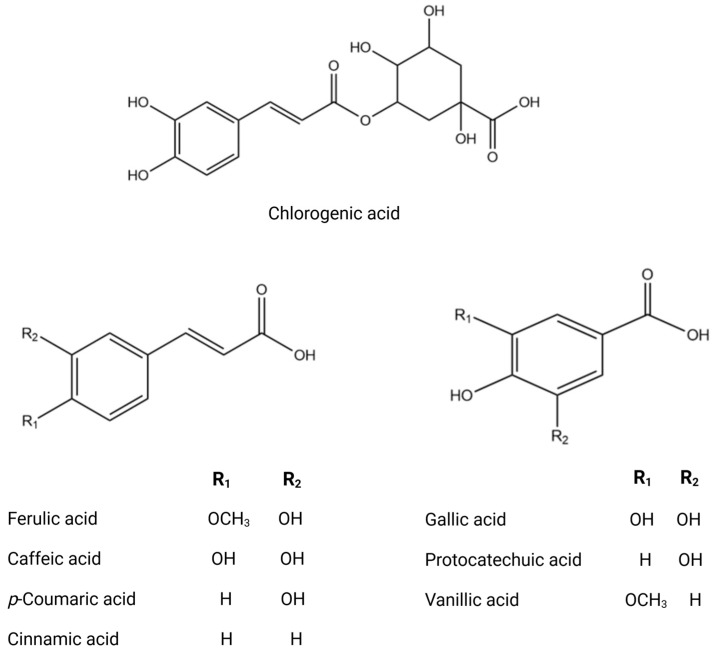
Chemical structures of key phenolic acids.

**Figure 4:**
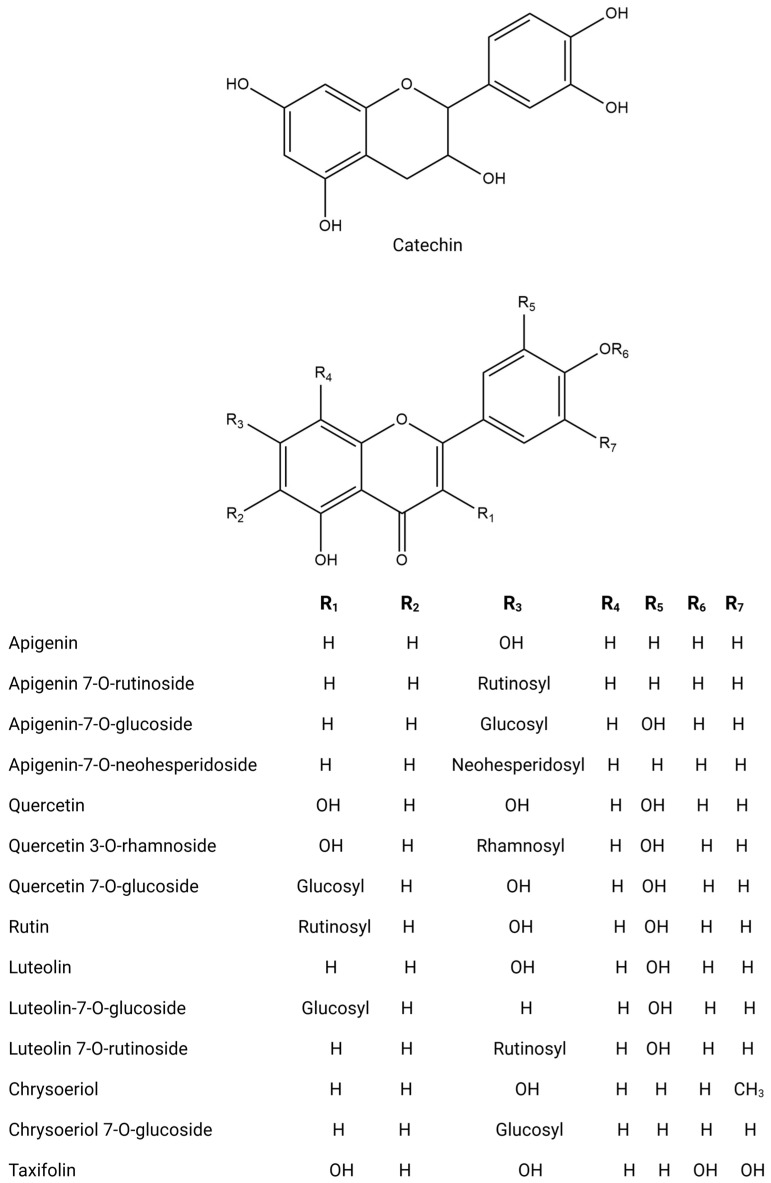
Chemical structures of key flavonoids.

**Figure 5:**
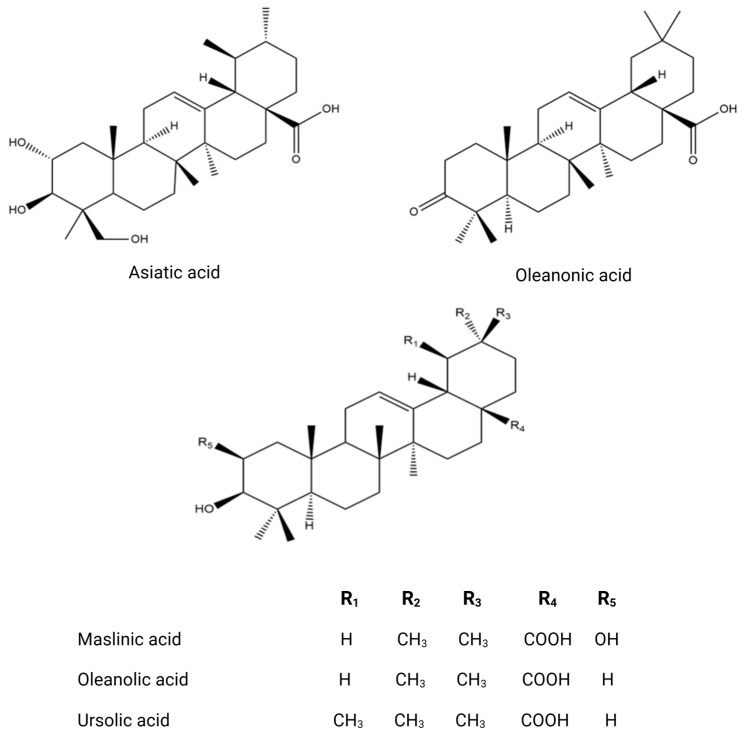
Chemical structures of key triterpenoids.

**Figure 6:**
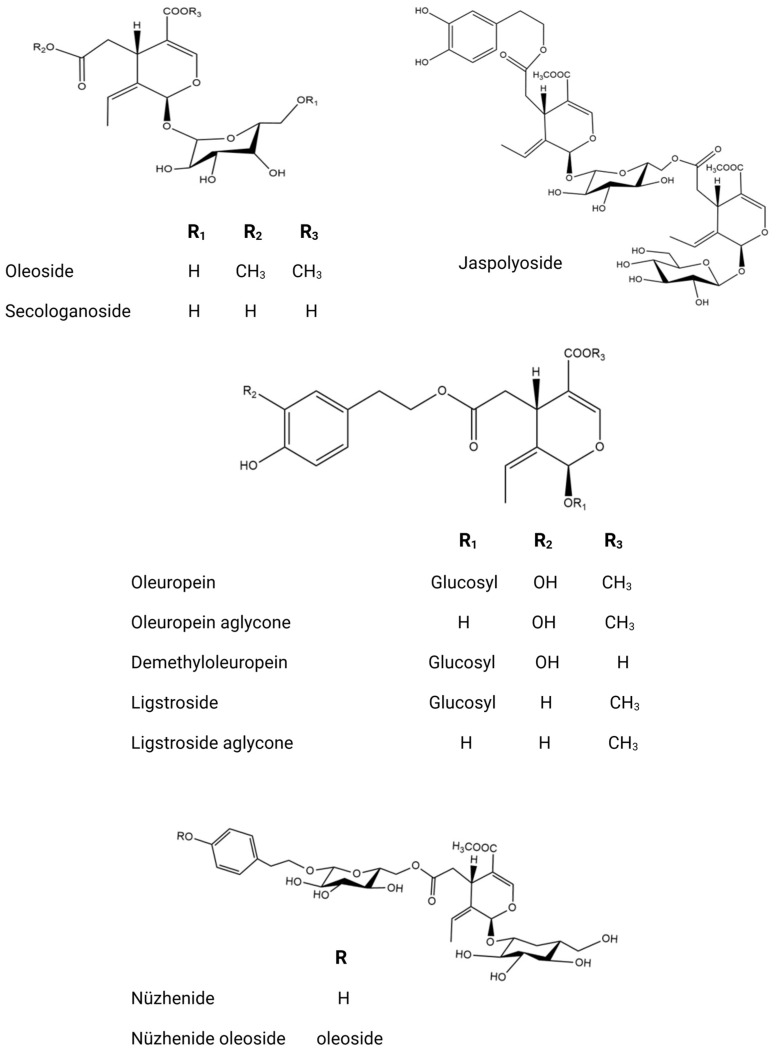
Comprehensive chemical structures of key secoiridoids.

The phytochemical research on *O. europaea* composition has revealed distinct profiles of secondary metabolites across various parts of the tree (Table 2). This species exhibits a wide range of biochemical complexity, encompassing triterpenoids, simple phenolics, iridoids, secoiridoids, and flavonoids, as illustrated in Figure 2, Figure 3, Figure 4, Figure 5 and Figure 6 [49]. Furthermore, the abundance of lipids, proteins, and minerals in its approximate composition highlights its potential as a great source of nutrition for human consumption.

Olive trees have a wide variety of bioactive chemicals in all their sections. Being a part of the *Oleaceae* family, *O. europaea* is distinguished by a particular subclass of phenolic chemicals called secoiridoids [60]. The ripening period of olives depends on geographic origin and the cultivation methods, which affect the concentration and quantity of secoiridoid distribution inside the olive tissues [76]. As a component of secondary metabolism, the production of oleuropein in *Oleaceae* involves a branching reaction inside the route of mevalonic acid, which eventually results in the generation of oleosides [77]. The stems and branches exhibit a high concentration of secondary metabolites, including triterpenoids like erythrodiol and maslinic acid, as well as phenolic substances like taxifolin, comselogoside, and oleuropein [52]. Numerous studies, including those by Talhaoui et al., (2014) and Cecchi et al., (2015) show support for this [9,78].

The fruit of the olive tree exhibits a significant diversity in both primary and secondary metabolites. Its vegetable oil is enriched with a variety of fatty acid compounds, predominantly polyunsaturated fatty acids, along with tocopherols and carotenoids, which play crucial roles in protecting against oxidative stress [54]. Additionally, olive oil is distinguished by the existence of volatile compounds such as isoprene, (*E*)-Hex-2-enal and *α*-copaene and other volatile molecules [79], as well as phenolic compounds including hydroxytyrosol, *p*-coumaric acid, quercetin, and luteolin. Numerous studies have highlighted the diverse chemical composition of olive oil [80,81]. The olive fruit possesses a valuable phenolic composition, characterized by flavonoids, secoiridoids, coumarins, phenolic acids, and triterpenoids, as evidenced by research [62,82,83]. Olive tree seeds are extremely interesting due to the wide variety of primary metabolites they contain, including proteins, fatty acids, and minerals [84]. One notable defining feature of olive seed oil is the presence of polar lipids, which include 94 lipid species and 10 polar lipid classes in their chemical makeup. Phospholipids, glycolipids, acylsterolglycosides, and sphingolipids are some of these classes [85]. When compared to saturated fats, the fraction of polyunsaturated fatty acids in these polar lipids is much larger [86]. Numerous investigations into the polar lipidome of olive seeds have identified notable variations across olive tree cultivars, which are ascribed to the existence or non-existence of certain lipid species. Polar lipid composition has been established and has attracted interest from the cosmetics and culinary sectors because of its significant nutritional significance [87]. Moreover, one fascinating finding about the seed’s phenolic compounds is that they belong to the subclass of secoiridoids, which is defined by the existence of nüzhenide and its derivatives (Figure 6). These compounds, which are exclusive to olive seeds, have strong biological activity and show great promise for improving human health [88]. The constituents of olive leaves, as reported from the essential oil extracted via hydrodistillation, exhibit an interesting chemical composition comprising oxygenated monoterpenes, oxygenated sesquiterpenes, and monoterpene hydrocarbons [61,89]. As secondary metabolites, sesquiterpenes have drawn significant attention in bio-pharmacology, particularly as potential natural anticancer agents [90]. An olive leaf extract contains a wide variety of phenolic chemicals, including phenolic acids (gallic acid, protocatechuic acid, vanillic acid, and salicylic acid); flavonoids (rutin, diosmetin, and apigenin); lignans (pinoresinol and syringaresinol); triterpenoids, secoiridoids, and derivatives (oleuropein, loganoside, and ligstroside) [91,92,93,94,95]. In light of the fact that olive leaves contain numerous bioactive molecules with varied biological functions, they have gained significant attention as potent by-products of the olive tree [96,97]. The phytochemical composition of the olive tree is highly diverse, resulting in various biological activities. Its bioactive molecules can act as antidiabetic, anti-inflammatory, antifungal, antibacterial, antioxidant, and anticancer agents [98,99,100,101,102].

### 3.5. Biological Properties

#### 3.5.1. Antibacterial Activity

Infections with bacterial pathogens have a significant impact on human pathology. Serious conditions can arise, such as *Helicobacter pylori* contributing to intestinal issues and *Mycobacterium tuberculosis* leading to respiratory tract diseases. Additionally, *Propionibacterium acnes*, and *Staphylococcus epidermidis* colonize the skin, resulting in similar opportunistic pathologies [103]. Numerous studies have showcased the antibacterial properties of olive extracts from the leaf, fruit, and seed. There are numerous screening results presented in Table 3 that demonstrate the evaluation of these properties using various methods, such as clinical trials, in vivo and in vitro testing.

The olive tree seed is characterized by specific sub-phenolic secoiridoids, notably nüzhenide and its derivatives [52]. Kadir et al. [104] investigated the amounts of flavonoid and phenolic chemicals, as well as the antibacterial, antioxidant, and antithrombotic capabilities of extracts produced from the seed, fruit, and leaf of the Halhali olive tree, which is planted in Hatay, Turkey’s Arsuz area. Their findings revealed that seed extracts exhibited antimicrobial activity, with minimum inhibitory concentration values ranging from 100 to 200 mg/mL against *Klebsiella pneumoniae*, *Pseudomonas aeruginosa*, *Staphylococcus aureus*, *Enterococcus faecalis*, and *Escherichia coli* [104]. The olive fruit possesses an intriguing chemical composition, as confirmed by numerous studies highlighting its rich bioactive composition [82]. Hanene et al. (2014) investigated a methanolic extract from olive oleaster fruit pulp, which demonstrated strong effectiveness against Gram-positive and Gram-negative bacteria, indicating non-selective antibacterial activity [105]. Guo et al. (2019) discovered that olive oil polyphenol extract (OOPE) inhibited the development of *L. monocytogenes*, with 1.25 mg/mL as the minimal inhibitory concentration (MIC). When compared to untreated cells, OOPE-treated cells showed a substantial drop (*p* < 0.05) in intracellular ATP concentrations, DNA levels or bacterial protein [102]. Other studies have also confirmed the antibacterial effects of olive oil [106,107]. It was suggested by Wang et al., (2017) that phenolic compounds affected the minor groove of genomic DNA, changing its secondary structure and morphology, in addition to increasing permeability of the cell membrane and inducing morphological changes in the cells [108]. Furthermore, the literature highlights the strong antibacterial activity demonstrated by olive leaf extract in numerous studies. Abdullah et al. [109] found that the heated olive leaf aqueous extract exhibited excellent antibacterial activity in opposition to other selected strains. Concentrations of the extract ranged from 0.5% to 6%, resulting in inhibition zone diameters ranging from 1 mm to 10.3 mm. Notably, *Staphylococcus aureus* and *Streptococcus mutans* demonstrated the greatest sensitivity to the extract. To elucidate the mechanism of action involving a mixture of polyphenols with absorption and bioavailability, agarose gel electrophoresis analysis of plasmid DNA isolated from kanamycin- and ampicillin-resistant bacterial strains was performed. The analysis aimed to determine if the olive leaf extract, rich in polyphenols, could interact with the plasmid DNA of the resistant bacterial strains. This interaction could potentially lead to the cleavage or alteration of the plasmid DNA, affecting the bacteria’s resistance mechanisms [109]. Alsulaymani et al. (2021) found that the extracts exhibited significant antibacterial activity against *Escherichia coli*, with inhibition zone measurements ranging from 8 mm to 15 mm [110]. De la Ossa et al. (2021) found that 100 mg/mL of olive leaf extract (OLE) showed antibacterial action; however, it was unable to completely inactivate the germs. Plain OLE decreased *L. innocua* and *S. aureus* growth, whereas *E. coli* growth was unaffected [111]. Himour et al. [112] noted that the variety of the olive tree and the choice of solvent in the extraction process significantly influenced the extraction of secondary compounds, consequently impacting extraction activity [112].

Furthermore, Rosa et al. [113] reported that microwave-assisted extraction (MAE) was more effective than ultrasonic-assisted extraction (UAE), inhibiting *E. coli* growth by 100% vs. 80%, respectively, at a dose of 50 mg/mL. Additionally, the inhibition effect of the UAE extract reached almost 92% at 75 mg/mL. The extraction process directly impacts the composition of the bioactive molecule extract, potentially altering its biological activity [113,114]. Vural et al. [115] found that the essential oil extracted from olive leaves displayed a highly intriguing volatile composition, showcasing potent antibacterial activity [115].

**Table 3:** A synopsis of research on olive tree extracts’ antibacterial activity.

Part Used	Extract	Strains	Method	Key Results	Reference
Seed	Vegetal extract	GP *Staphylococcus aureus* *Enterococcus faecalis* *Listeria innocua* *Listeria monocytogenes* *Bacillus cereus* *Streptococcus mutans* *Bacillus subtilis* *Staphylococcus epidermidis* *Clostridium sporogenes* *Bacillus subtilis subsp. Spizizenii* *Bacillus subtilis* *Streptococcus sobrinus* *Streptococcus ralis* *Staphylococcus epedermidis,* *Propionibacterium acnes* *Lactobacillus plantarum* GN *Escherichia coli* *Salmonella typhimurium* *Aeromonas hydrophila* *Agrobacterium tumefaciens* *Pseudomonas aeruginosa* *Acinetobacter baumannii* *Shigella. sonnei* *Proteus mirabilis* *Citrobacter freundii* *Salmonella. enteritidis* *Pseudomonas fluorescens* *Brochotrix thermosphacta* *Pseudomonas fragi* *Pseudomonas putida* *Salmonella enterica* *Enterobacter cloacae* *Klebsiella pneumoniae* *Salmonella Enteritidis* *Pseudomonas vulgaris* *Morganella Morganii* *Haemophilus influenzae* *Yersinia enterolitica Salmonella enterica subsp. heindelberg*	Disc diffusion method Minimum inhibitory concentration Minimum bactericidal concentration Broth micro-dilution assay Live/dead bacterial staining assay Bacterial inhibition assays Bacterial motility assays	MIC = 100–200 µg/mL	[104]
Fruit	Vegetable oil			Ø = 5–18 mm	[81,116]
	Vegetal extract			Ø = 13–18.5 mg/mL MIC = 12.5–25 mg/mL MBC = 25–50 mg/mL	[102,105]
Leaves	Essential oil			Ø = 9–29 mm MIC = 0.625–5 mg/mL	[6,96,115,117]
	Vegetal extract			Ø = 1–20 mm MIC = 0.60–25 mg/mL MBC = 0.70–12.5 mg/mL	[109,110,118,119,120,121,122,123,124,125,126,127]

Ø = zone of inhibition; MIC = minimum inhibition concentration; MBC = minimum bactericidal concentration; GN = Gram negative; GP = Gram positive.

#### 3.5.2. Antifungal Activity

Microbial infections have emerged as a significant global concern, with the *Candida albicans* species being one of the most prevalent fungal strains found on human mucosal membranes [126]. This yeast can attach to epithelial tissues, resulting in superficial infections commonly referred to as candidiasis. Under certain conditions, candidiasis has become a major contributor to morbidity and mortality among immunocompromised individuals worldwide [128]. A primary challenge lies in the multidrug-resistant nature of both bacterial and fungal strains [129,130]. Consequently, researchers are increasingly turning to phytotherapy using medicinal plants, which possess antimicrobial properties due to their chemical compounds. However, the diversity of extraction techniques for bioactive molecules poses a critical challenge to their efficacy against fungal infections. *O. europaea*, a medicinal plant, is known for its potent microbial-infectious molecules due to its phenolic composition, particularly secoiridoids [131]. Several studies have investigated the biological activity of various portions of the olive tree, as shown in Table 4. Olive leaves, in particular, are characterized by their phenolic composition, which exhibits potent antifungal properties [132]. Vural et al. [115] assessed the antimicrobial effects of dried leaf essential oil towards microbial strains, and discovered considerable antifungal activity against *Candida albicans* [115]. Similarly, other researchers observed intriguing effects of leaf essential oil against fungal infections [133]. However, several factors can affect the effectiveness of these extracts, such as the variety of olive cultivar, the extraction process, the solvent used, and the by-product harvesting time. Alimosazade et al. [134] examined olive extracts from four cultivars against various fungal strains. The minimum inhibitory concentration (MIC) varied due to differences in total phenolic content, indicating the impact of cultivars on biological activity efficacy [134]. Bawadekji et al. [135] performed analyses on the phytochemical composition and antimicrobial properties of olive leaf extracts obtained using water-immiscible solvents, observing significant inhibition of *Aspergillus niger* and *Candida albicans* growth [135]. This study emphasized the impact of extraction solvent on antifungal activity. Overall, olive leaf extracts exhibited substantial antifungal activity attributed to their rich phenolic compound content, particularly hydroxytyrosol, oleuropein, and secoiridoid derivatives, consistent with findings from several studies [136,137,138]. The olive fruit is known for its rich array of bioactive molecules that possess antimicrobial properties [139]. Naje et al. [140] investigated the *O. europaea* fatty oil’s possible antifungal action against clinical isolates of fungal strains, yielding promising results as presented in (Table 4) [140]. Similarly, Janakat et al. [141] reported a favorable MIC of olive fruit oil against fungal growth [141]. Additionally, Umai et al. [142] produced silver nanoparticles from olive fruit extract as an environmentally sound approach to demonstrate effective MIC values against tested fungal strains [142]. Suwan et al. [143] also highlighted the noticeable activity of silver nanoparticles synthesized from olive extract against fungal species [143]. Biofilm-associated infections, accounting for up to 80% of total human infections, pose significant challenges to the medical community and protect microbial cells from the host immune response [144]. Slobodnikova et al. [14] revealed the significant activity of phenolic compounds from olive fruit against biofilms formed by bacteria and fungi species [14]. Furthermore, nanoparticle-based treatments derived from olive extracts exhibited powerful antibiofilm activity [145]. Several research works have examined the chemical makeup of olive seeds and their antifungal activity, with olive seed extracts demonstrating activity against various fungal strains [146]. The bioactive compounds of the olive tree are strongly correlated with the efficacy of our extracts against fungi.

#### 3.5.3. Antioxidant Activity

Reactive oxygen and nitrogen species (RONS) are chemically active free radicals and non-radical substances produced during normal cellular processes and mitochondrial respiration, including ozone (O_3_), hydrogen peroxide (H_2_O_2_), hypochlorous acid (HOCl), peroxynitrite ion (ONOO), and singlet oxygen (O_2_) and oxygen radicals like hydroxyl (HO), superoxide anion (O_2_), peroxyl (ROO), hydroperoxyl (HO_2_), and alkoxyl (RO). Correspondingly, reactive nitrogen species (RNS) comprise nitric oxide radical (NO), nitrogen dioxide radical (NO_2_), peroxynitrite ion (ONOO), and certain nitrogen oxides [151,152]. To manage these reactive species, the body employs endogenous enzymes like catalase, glutathione peroxidase, and superoxide dismutase [153]. Yet, under conditions of increased stress or physiological imbalance, the generation of RONS might exceed the capabilities of the body’s internal antioxidant mechanisms, resulting in oxidative stress. The structure or functionality of biological macromolecules such as proteins, lipids, and DNA may change as a result of this stress [154,155]. Interestingly, the oxidation of macromolecules in cells is believed to be a significant factor in the development of various age-related and degenerative diseases [156,157,158,159]. Considerable research has focused on exploring natural sources of bioactive molecules found in medicinal plants to address the imbalance of ROS within the human body. Among these efforts, there has been extensive investigation into the effects of the chemical composition of *O. europaea* in combating oxidative stress. Table 5 summarizes the findings from this research.

**Figure 7:**
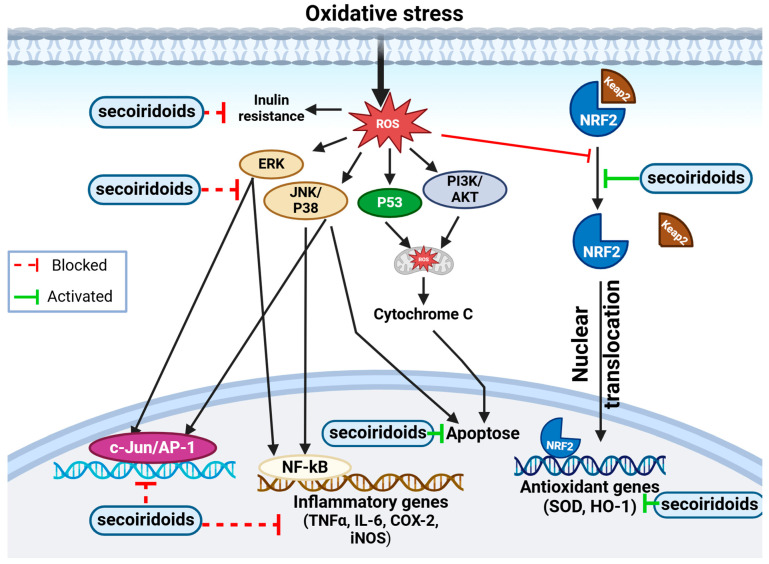
Figure illustrating the molecular representation of the antioxidant mechanism of secoiridoids. This potent antioxidant has the potential to mitigate intracellular ROS levels. ROS, known to induce inflammation via NF-kB and AP-1 activation, trigger various cellular signaling processes leading to the production of inflammatory mediators. Secoiridoids effectively block these mediators. Additionally, secoiridoids might regulate cellular signaling by impacting signal transduction pathways. They demonstrate protective properties by activating the PI3-kinase/Akt pathway, stimulating MAPK proteins (ERK, JNK, and P38), and facilitating Nrf2 translocation into the nucleus. Furthermore, secoiridoids elevate the expression of MnSOD and HO-1, providing defense against oxidative stress through the Nrf2 pathway.

Kadir et al. [96] conducted a comprehensive exploration of the potential for pharmaceuticals of extracts from the Halhali olive’s leaf, fruit, and seeds using various methodologies. Their investigation revealed significant antioxidant activity in the extracts. Specifically, with 317.24 μg GAE, the seed extract had the highest phenolic content and demonstrated the strongest reducing potential for Fe^3+^/Fe^2+^. Additionally, it displayed notable radical scavenging activity against DPPH, with an IC_50_ value of 5.25 µg/mL [96]. Celik et al. [99] reported that aqueous and ethanolic olive seed extracts exhibited significant antioxidant activity against DPPH, ABTS and H_2_O_2_ radicals at a concentration of 30 µg/mL. The high phenolic and flavonoid content of both extracts may account for this strong activity [99]. Notably, Bouarroudj et al. [99] reported that oleaster oil exhibited the richest phenolic composition among all samples studied. They identified two new molecules, namely eriodictyol and naringenin. In addition, the antioxidant potential of olive oil against DPPH and ABTS radicals showed significant effects, with 80% inhibition of free radicals in both tests. The phenolic composition and amount of tocopherol in olive oil are responsible for its greater inhibitory capacity [160]. Fernández-Poyatos et al. [167] studied the antioxidant capacity and phenolic content of table olives and the effect of in vitro simulated digestion. They stated that the extract from table olive fruit contained a significant amount of phenolic compounds, comprising thirty different compounds. The in vitro digestion assay revealed a reduction of over 50% in total phenolic content, thereby impacting antioxidant potency. Both antioxidant assays (ABTS and DPPH) showed a substantial decrease in activity. The digested assay offers an initial insight into the potential of these compounds to traverse the digestive system. The bioavailability of these molecules is key to optimizing their protective effects [167]. Furthermore, Tamasi et al. [168] aimed to investigate the *O. europaea* products’ chemical composition and antioxidant capabilities; they found that olive fruit extract exhibited a high level of total phenolic content (TPC), with quantitative variations influenced by factors like olive variety, harvest time, soil, and climatic conditions. The antioxidant assays (DPPH and ABTS) showed correlated results with TPC, indicating that higher TPC correlated with increased antioxidant potential [168,169]. Moreover, numerous studies have underscored the significant antioxidant role of olive leaves. Elnahas et al., (2021) found that Egyptian olive leaf extracts exhibited 87.55% scavenging activity against DPPH free radicals at a concentration of 50 mg/mL [119]. Blasi et al. [170] examined the seasonal variations in the antioxidant components of *O. europaea* leaf extracts from different Italian cultivars. They observed that seasonal variation significantly influenced the antioxidant potential of the olive leaf extract and the TPC. Moreover, the predominant phenolic content and components are at the origin of the extract’s antioxidant capacity [171]. These findings suggest that a higher TPC in a season does not necessarily correlate with the highest antioxidant capacity. For instance, DPPH inhibition ranged from 55% to 86%, and ABTS ranged from 53.6 to 88.4 mg TE/g [170]. Farag et al. [7] conducted phytochemical screening and assessed the crude juices’ antioxidant content from various medicinal plants. They observed that the antioxidant capacity of the olive leaf extract had a positive effect compared to the standard (std) used. In the DPPH assay, the IC_50_ was 42.5 µg/mL, while the std/IC_50_ was 21 µg/mL. For metal chelating activity, the EDTA/IC_50_ was 155 µg/mL, and the std/IC_50_ was 37.95 µg/mL. Raw olive leaf juice is ideally suited to practical use as a dietary supplement to delay fat oxidation [7]. Lins et al., found that olive leaf extract inhibited DPPH (EC_50_ = 13.8 ± 0.8 mg/mL) and ABTS (EC_50_ = 16.1 ± 1.2 mg/mL), and FRAP analysis produced a value of 281.8 ± 22.8 mg TE/g dry weight. The extract effectively scavenged HOCl (EC_50_ = 714.1 ± 31.4 mg/mL), NO (EC_50_ = 48.4 ± 6.8 mg/mL), and O_2_ (EC_50_ = 52.6 ± 2.1 mg/mL). In addition, it prevented erythrocytes from undergoing the action of peroxyl radicals that produce hemolysis (EC_50_ = 11.5 ± 1.5 mg/mL), TBARS formation (EC_50_ = 38.0 ± 11.7 mg/mL) and hemoglobin oxidation (EC_50_ = 186.3 ± 29.7 mg/mL). These findings indicate that olive leaf extract is a remarkable, natural antioxidant source, exhibiting potent antioxidant activity against a range of reactive species and protecting human erythrocytes against free radical damage [172]. Furthermore, Ayoub et al. [173] conducted a phytochemical screening and assessed the antioxidant activity and inhibitory capacity of *O. europaea* and *Ficus carica* leaves. They observed that olive leaf extract exhibited robust antioxidant potency in scavenging free radicals, with a relationship between antioxidant activity and secoiridoids and flavonoid contents (Figure 7). The IC_50_ value of the *O. europaea* extract was determined to be 170.134 ± 0.06 μg/mL, indicating its efficacy in inhibiting DPPH free radicals and mitigating oxidative stress [173]. Lfitat et al. [163] conducted in vitro antioxidant studies to confirm the antioxidant potency of olive leaves and their presence in secondary metabolites, which aid in metal chelation and free radical scavenging [163]. Monteleone et al. also confirmed that olive leaf extract (OLE) displayed significant antioxidant capacity. At peak levels of phenolic and flavonoid content, the extract demonstrated the most potent inhibitory effect on oxidation, with a DPPH = 2.85 ± 0.01 TEAC mM for 90% inhibition [18,174]. Ribas et al. assessed the nutritional composition, bioactive components, antioxidant properties, and color characteristics of leaves sourced from eight different olive cultivars in Brazil. They found that the olive leaf extract (OLE) from these varieties exhibited the greatest level of antioxidant capability, with ABTS = 78.15% and DPPH = 93.56%. Several factors have been identified that influence the content of secondary metabolites and antioxidant activity, including olive cultivar, date of collection, drying conditions, climatic conditions and extraction method [175,176,177]. Furthermore, Pennisi et al. [165] sought to assess the antiviral and antioxidant properties of leaf extracts obtained from *O. europaea* vars. sativa and sylvestris. Researchers discovered that exposure to olive leaf extracts prevents lipid peroxidation in human HeLa cells and boosts the levels of antioxidant enzymes including catalase, superoxide dismutase, and glutathione peroxidase, which in turn lessens the oxidation of free radicals [165].

#### 3.5.4. Antidiabetic Activity

Diabetes mellitus (DM) and its treatment represent considerable social, economic, and healthcare challenges on a global scale. DM is a metabolic disorder characterized by chronic hyperglycemia, along with imbalances in lipid, protein, secretion, and insulin action [178]. Projections suggest that by 2025, over 300 million people will be affected by diabetes [179]. Type I diabetes arises from defects in insulin secretion due to inherited and/or acquired deficiencies in pancreatic insulin production [180]. On the other hand, type II diabetes results from insulin ineffectiveness caused by insulin resistance in the liver and peripheral tissues [181]. As type II diabetes advances, there is a decrease in cell mass and function, disrupted insulin signaling, changes in lipid metabolism, low-grade inflammation, and heightened oxidative stress [182]. Diabetes pathogenesis and complications are greatly influenced by oxidative stress. It induces injury in pancreatic cells and enzymes, increases lipid peroxidation, and leads to insulin resistance in cells [15]. There has been strong evidence that *O. europaea* may be used as an ethnomedicine in the treatment of diabetes, as illustrated in Table 6 and Table 7. Notably, Perri et al. [183] found that *O. europaea* bud extracts inhibited pancreatic lipase and *α*-amylase activity in several Italian cultivars. The study found significant suppression of digestive enzymes, with IC_50_ values of 33.21 ± 0.23 µg/mL for *α*-amylase and 1.27 ± 0.04 mg/mL for lipase inhibition [183]. *O. europaea* subsp. *cuspidata* (Indian olive) seed extracts were studied by Akhtar et al., (2022) for their antioxidant activities and potential as antidiabetic agents. At a concentration of 1.6 mg/mL, in vitro tests revealed an 82.10% inhibition of *α*-Amylase. Rats were used in in vivo tests to generate diabetes with a single intraperitoneal quantity of alloxan monohydrate (150 mg/kg). Three days following the injection of alloxan, rats’ serum FBG levels were assessed; those rats whose FBG levels were 200 mg/dL were classified as diabetic. Rats without diabetes were given normal saline orally in Group 1, while diabetic rats were given glimepiride (0.2 mg/kg) orally in Group 2, which served as the standard control. Rats with diabetes who were given normal saline alone made-up Group 3, the negative control. The application of MEOE resulted in a dose-dependent recovery of the Langerhans islets, reduced kidney blood vessel inflammation and thrombosis, and lessened glomerulus and tubule necrosis caused by alloxan [184]. Furthermore, olives are rich in biophenol compounds, providing them with the ability to combat various biological diseases. Zakari et al. [185] worked on the hypoglycemic effect of olive oil on alloxan-induced diabetic albino rats. Their study revealed that olive oil demonstrated a hypoglycemic effect on alloxan-induced diabetic Albino rats at a lower dose (150 mg/kg/b.w.) [185]. Figueiredo-González and colleagues [186] examined the potential antidiabetic effects of phenolic-rich extracts derived from autochthonous extra virgin olive oils in Galicia towards the inhibition of *α*-amylase and *α*-glucosidase. They discovered that EVOO had a stronger antidiabetic impact on *α*-glucosidase (IC_50_ = 0.06 ± 0.008 mg/mL) than it did on *α*-amylase [186]. Additionally, Abdelkarim et al. [187] studied the effects of olive leaf powder (OLP) on circulating adipokines and insulin secretion in rats with streptozotocin-induced diabetes. Four groups (n = 10) of 40 male albino rats from Wistar weighing 200–225 g each were created: normal healthy rats were included in Group I; diabetic control rats were included in Group II, diabetic rats, fed a balanced diet along with standard antidiabetic medication (metformin, 600 mg/b.w.), were included in Group III, and diabetic rats were included in Group IV, given a diet plus 2.0% OLP. After analyzing their data, they concluded that OLP significantly improved blood glucose regulation. It did this by lowering serum levels of glucose, low-density lipoprotein, triglycerides, and total cholesterol, elevating levels of high-density lipoprotein; decreasing levels of atherogenic index and atherogenic coefficient; raising serum adiponectin concentration; and lowering serum leptin concentration [187]. Al-Shudiefat et al. [188] sought to investigate the mechanism behind this phenomenon by assessing proteins involved in glucose metabolism, including adenosine monophosphate-activated protein kinase (AMPK α2), glucose transporter 4 (Glut4), and Akt substrate of 160 kDa (AS160), in an effort to understand the mechanism behind this behavior. Their findings revealed that treatments with OLE at 1% and 3% effectively reduced blood sugar levels and regulated biochemical parameters such as LDL, HDL, TG, and insulin secretion. They observed that OLP inhibits AS160, resulting in decreased blood glucose levels [188]. Chigurupati et al. [189] found that OLE exhibited a significant *α*-amylase inhibitory effect, with an IC_50_ of 0.037 µg/mL, demonstrating competitive kinetic inhibition [189]. Additionally, Laaboudi et al. [190] sought to investigate the hypolipidemic and hypoglycemic properties of olive tree extract phenol on streptozotocin-induced diabetic rats. They discovered that taking olive extract orally led to a decrease in blood glucose levels in the diabetic rat group, which were significantly lower at 4 weeks compared to control diabetic rats. Furthermore, the extract regulated the biochemical parameters to approximate those of normal rat models [190].

**Table 6:** Antidiabetic activities of various parts of *O. europaea:* summary of in vitro research findings.

Part Used	Extract	Method In Vitro	Key Results	Reference
Buds	Hydroalcoholic extract	Pancreatic lipase activity inhibition (PLI) The inhibition of *α*-amylase (IAM) The inhibition of *α*-glucosidase Glucose uptake using the yeast cells assay Determination of surface GLUT4myc L6-GLUT4myc cell line	IC_50_ = 1.27 ± 0.04 mg/mL IC_50_ = 0.1269 ± 0.023 mg/mL	[183]
Seed	Aqueous extract		IC_50_ = 0.3194 mg/mL	[184]
Fruit	Ethyl acetate extracts		IC_50_ = 0.00531 ± 0.003 mg/mL IC_50_ = 0.0547 ± 0.001 mg/mL	[191]
	Extra virgin olive oil		IC_50_ = 0.06 ± 0.008 mg/mL	[186]
Leaves	Hydroalcoholic extract		IC_50_ = 150 µM	[101]
	Ethanolic extract		IC_50_ = 0.037 mg/mL	[189]
	Aqueous extract		IC_50_ = 0.014 ± 0.041 µg/mL	[17]
			IC_50_ = 0.2 mg/mL	[192]
	Methanolic extract		IC_50_ = 43.47 µM EC_50_ = 47.12 µM	[193]

Mansour et al. [17] showed that administering OLE alone or combined with metformin normalized blood glucose, glycated hemoglobin, lipid profiles, and liver enzyme levels. Histological analysis indicated that OLE, either alone or combined with metformin, effectively restored liver, kidney, and pancreatic tissues [17]. Mechchate et al. [16] explored the in vivo antidiabetic effects and the in silico mode of action of flavonoids found in the leaves the Oleaster using LC/MS-MS analysis. They found that the flavonoid extracted from olive leaves, in two concentrations (25–50 mg/kg/b.w.), effectively managed diabetes induced by alloxan in in vivo experiments. It demonstrated a strong regulation of biochemical parameters, with this effect being even more pronounced when coupled with an antidiabetic drug [16]. Rauf et al. [193] investigated the antidiabetic effect of Ferruginan using the yeast cell glucose uptake assay. At the highest tested dose (100 μM), Ferruginan showed a maximum reduction in inflammation of 71.82% and improved absorption up to 74.96% of glucose by yeast cells. Additionally, Ferruginan dose dependently inhibited *α*-amylase, showing inhibition of up to 75.45% at the same concentration [193]. Therefore, the extract from olive leaves has the potential to exhibit inhibitory effects on *α*-amylase and *α*-glucosidase, similar to acarbose, due to the presence of flavonoids, secoiridoids and other bioactive compounds [194].

**Table 7:** Antidiabetic activities of various parts of *O. europaea:* summary of in vivo research findings.

Part Used	Extract	Dose	Method In Vivo	Key Results	Reference
Seed	Methanolic extract	750 mg/kg body weight	Alloxan-induced diabetes in Wistar albino rats Swiss albino mice (aged 3–4 weeks) of both sex Wistar albino male rats (streptozotocin) Sprague–Dawley male rats (streptozotocin)	The treatment of seed MeOH extracts was seen to have a significant hypoglycemic impact and to have reversed weight loss in diabetic rats.	[184]
Fruit	Vegetable oil	150 mg/kg body weight		The blood sugar levels of the group 2 alloxan-induced diabetic rats exhibited a gradual decline, eventually stabilizing within the normal range of 4.9–5.5 mg/dl.	[185]
Leaves		2%/kg body weight		Low-density lipoprotein, total cholesterol, triglycerides, and serum glucose levels were all lowered. High-density lipoprotein levels rose as a result. It reduced atherogenic index and atherogenic coefficient.	[187]
	Aqueous extract	200–400 mg/kg body weight		The application of both low and high doses of olive leaf extract effectively ameliorated the identified physiological, molecular, and histopathological changes.	[195]
		1–3%/kg body weight		Lowered blood glucose levels, regulated biochemical parameters including LDL, HDL, TG, and insulin secretion. OLP functions by inhibiting AS160, thereby causing a reduction in blood glucose levels.	[188]
		100 mg/kg body weight		Oral treatment with olive extract controlled biochemical parameters to mimic normal rat models and contributed to a considerable reduction in blood glucose levels in the diabetic rat group after 4 weeks when compared with control diabetic rats.	[190]
	Methanolic extract	25 and 50 mg/kg body weight		It demonstrated a strong regulation of biochemical parameters, and this effect was even more pronounced when coupled with an antidiabetic drug.	[196]
		25 mg/kg body weight		The flavonoid extracted from olive leaves had a positive effect on the blood glucose level, showing a significant reduction of 49.59% compared to the normal control group.	[16]

#### 3.5.5. Anticancer Activity

Cancer, a multifaceted chronic degenerative ailment, involves a multistep transformation where normal cells become malignant, displaying aberrant proliferation and diminished apoptosis [197]. Advances in cancer molecular understanding have uncovered key targets for anticancer drug development and treatment strategies [198]. Biophenols have emerged as agents capable of modulating cell growth at different stages of cancer development by either triggering apoptosis or restraining proliferation through a variety of mechanisms [199,200]. Constituents of *O. europaea* exhibit potent anticancer properties against various types of cancer [201]. The summarized results of various studies are reported in Table 8. Additionally, Xie et al. [50] reported that the seed extract from the Frantoio olive cultivar demonstrated the least cytotoxicity on colorectal cells, as evidenced by its high IC_50_ value. Triterpenoids, notably ursolic acid found in the seed extract, have been shown to inhibit proliferation in colorectal cancer cells (HCT-116). This study evaluated the cytotoxic effects of the seed extract on HCT-116 colorectal cells, providing valuable insights into its potential for cancer therapy [50]. Carpi et al. [202] aimed to investigate the in vitro antimelanoma activity of OA and its mechanism of action. Their findings revealed that oleacein (OA) inhibited cell growth in melanoma cells with an IC_50_ in the low micromolar range, showing time- and concentration-dependent effects. In melanoma cells, it caused DNA fragmentation, G1/S phase arrest, and downregulation of genes producing proliferative and antiapoptotic proteins. The overt antigrowth activity of OA in melanoma cells (501 Mel) occurs at low micromolar concentrations, suggesting specific targeting mechanisms that are hyper-reactive in cancer [202]. Additionally, Maalej et al. [203] sought to examine the phytochemical composition of ethanolic extracts from olive fruits of three distinct cultivars (OFE) and explored their antioxidant properties and potential anticancer effects. Their study revealed that after 48 h of cultivation, flow cytometry analysis demonstrated that OFE caused cell cycle arrest in the S-phase in both Caco-2 cells and HepG2. The IC_50_ of OFE was higher in HepG2 cells compared to Caco-2 cells, indicating that the olive fruit extract was more sensitive against the Caco-2 cell line than the HepG2 cell line [83]. Quero et al. [204] investigated the hydroethanolic extract of olive pomace, revealing its diverse polyphenolic composition. Their study showed that the extract significantly altered the mitochondrial membrane during a 72 h cell culture, which resulted in cell cycle arrest in the G1/S stages and death. In the Caco-2 cell line, this mechanism involves the activation of the proteins caspase 3 and p53 [204]. Albogami and Hassan [205] investigated the anticancer properties of an olive leaf extract in vitro using colorectal (HT29) and prostate (PC3) cancer cell lines. In both cell lines, the aqueous olive leaf (AOL) extracts stopped cells in the S phase. Moreover, they decreased cell migration and displayed multiple apoptotic characteristics, such as nuclear fragmentation, microtubule protrusions, apoptotic bodies, and blebbing. Additionally, the AOL extract induced DNA fragmentation in both cell lines [205]. Uğuz et al. [206] showed that when tested against the HL-60 leukemic cell line, olive leaf extract (OLE) had strong cytotoxicity. Remarkably, the removal of chlorophyll pigments significantly diminished the cytotoxic effect in both cases. This suggests that chlorophylls play a crucial role in the tumor-killing ability of such plant-derived extracts [206]. Notably, Shah et al. [207] investigated the *Olea ferruginaea* bark ethyl acetate extract, resulting in the identification of two previously known chemicals, ferruginan (2) and cycloolivil (3), as well as a new compound, ferruginan A (1). These substances were examined in tests using the MCF-7 breast cancer cell line. Compounds 1–3 showed modest cytotoxicity (IC_50_ = 8.03–12.01 μg/mL) in comparison to the standard (IC_50_ = 4.41 μg/mL) against MCF-7 cells, whereas the ethyl acetate fraction had high cytotoxic activity (79.31% inhibition at 250 μg/mL). This demonstrates that the separated chemicals have a modest anticancer effect [207]. Samet et al. [208] investigated the effects of olive leaf extract (OLE) on human chronic myelogenous leukemia K562 cells, focusing on apoptosis induction and monocyte/macrophage differentiation. After 72 h of treatment with 150 μg/mL of OLE, cell proliferation was inhibited to 17% compared to the control cells. However, the mechanisms underlying its differentiation-inducing properties remain poorly understood [208]. Moreover, using four cancer cell lines (breast, colon, hepatocellular, and cervical carcinoma cells), Morsy and Abdel-Aziz [209] examined the anticancer properties of the methanolic extract of olive leaves. The results were compared with those of the standard drug vinblastine. The findings demonstrated the cytotoxic activity of olive leaf extract against various cancer cell lines. The IC_50_ values of the methanolic extract from olive leaves against HCT, MCF-7, HELA, and HEPG-2 cancer cell lines were determined as 81.6, 43, 21.5, and 77.9 mg GAE/L, respectively. These findings highlight the strong impact of the olive leaf extract on the HEPG-2 cancer cell line, meeting the cytotoxicity criterion for crude extracts established by the US National Cancer Institute, which is an IC_50_ < 30 mg/L [209].

#### 3.5.6. Toxicology Investigation

Wistar rats were used in the examination of the acute and subacute oral toxicities of the ethanolic extract of olive leaves (EEO) by histopathological analysis and the assessment of biochemical and hematological markers. Acute toxicity was evaluated through a single oral gavage of 2000 mg/kg of EEO in male and female rats. Subacute toxicity was assessed by administering different doses (100, 200, and 400 mg/kg) of EEO to male and female rats for 28 days via oral gavage. Animals were divided into four groups of ten (five males and five females), and their body weights were recorded during the treatment period. Liver and kidney samples were histopathologically analyzed post-euthanasia, involving routine processing, paraffin embedding, sectioning, and staining with hematoxylin and eosin. Hematological and biochemical parameters were measured, and histological analysis was performed by a trained histologist [22]. During the acute oral administration test, administering a dose of 2000 mg/kg of EEO did not lead to any mortality or signs of toxicity throughout the treatment period. Notably, there were no notable variations in body weight between male and female subjects, and there were no observable alterations in behavior among the exposed animals. Although certain hematological parameters, such as HGB, CHCM, HCT, RBC, MCV, and PLT, differed significantly from the control group (both males and females), they remained within the normal range. Long-term exposure to EEO at doses of 100, 200, and 400 mg/kg was tested for subacute toxicity, and the results showed no differences between the treatment and control groups in the measured hematological parameters (HGB, CHCM, HCT, RBC, MCV, PLT, and WBC). Nevertheless, the blood concentration of BUN notably rose in male subjects exposed to EEO at doses of 100 and 400 mg/kg compared to the control group. Importantly, histological analysis of liver and kidney tissues revealed no abnormalities following treatment with EEO in rats for both experiments [22]. Another study aimed to investigate the toxicological safety assessment of an extract of *O. europaea* leaves (Bonolive™). The results of the study showed no evidence of mutagenicity in an in vitro mammalian chromosomal aberration test and a bacterial reverse mutation test. Moreover, in an in vivo mouse micronucleus test, no genotoxic effects were found, even at doses that reached the maximal dosage of 2000 mg/kg/bw/d. In a 90 d repeated oral toxicity assessment, Bonolive™ did not induce mortality or adverse effects in Crl:(WI) BR Wistar rats at doses of 360, 600, and 1000 mg/kg/bw/d. The highest dose tested, 1000 mg/kg bw/d, was determined as the no-observed adverse-effect level for both male and female rats in the 90 d study [216]. Additional randomized controlled human clinical studies along with comprehensive toxicity evaluations are required to identify any health effects and ensure safety.

## 4. Conclusions and Prospects

In conclusion, this review outlines the usage of *O. europaea* in ethnobotany, phytochemistry, pharmacology, and toxicological studies. Found extensively across various countries, this plant is utilized by diverse populations for treating specific ailments. Ethnobotanical surveys demonstrate a wide range of uses influenced by the plant part utilized, targeted disease, and geographical region. Phytochemical analysis revealed that *O. europaea* contains secoiridoids and various chemical compounds from classes like terpenoids, flavonoids, and phenolic acids. Pharmacological assays have scientifically validated the traditional uses of *O. europaea*, correlating ethnopharmacological applications with its biological activities and secondary metabolite content. Additionally, both in vitro and in vivo studies on *O. europaea* essential oils, vegetable oils, and extracts have revealed various effects, including antibacterial, antifungal, antioxidant, antidiabetic, and anticancer activities. Toxicological evidence in animal models did not reveal significant toxicity. The published data generally show consistent and satisfactory results, but knowledge remains limited, particularly in clinical trials. Through more preclinical and clinical research into illnesses associated with reactive oxygen species (ROS), more study is needed to clarify the biochemical and biological activities, as well as the pharmacokinetics and pharmacodynamics, of secoiridoids from olive trees.

## Figures and Tables

**Table 1:** Charting the worldwide dispersion of olive varieties.

Country	Olive Variety	Reference
Albania	Kalinjot	[29]
Algeria	Blanquette de Guelma, Azeradj, Sigoise	[30]
Argentina	Arauco	[31]
Chile	Azapa	[32]
Cyprus	Ladoelia, Kato Drys	[33]
Croatia	Oblica, Crnica, Karbonaca	[34]
Egypt	Toffahi, Koroneiki, Aggezi Shami	[35]
France	Grossane, Aglandau, Picholine, Tanche	[36]
Greece	Koutsourelia, Kalamón, Megaritiki, Mastoidis	[37]
Israel	Kadesh, Barnea, Merhavia	[38]
Italy	Leccino, Pisciottana, Frantoiana, Salella, Ravece, Cilentana, Carolea	[39]
Jordan	Rasi’i	[40]
Lebanon	Soury, Maurino, Baladi	[41]
Morocco	Picholine Marocaine, Menara, Haouzia, Arbequina	[42]
Palestine	Nabali Baladi, Yunani, Barouni	[43]
Portugal	Maçanilha Algarvia, Cordovil de Serpa, Galega, Carrasquenha, Redondal	[44]
Spain	Lechín de Sevilla, Manzanilla de Sevilla, Picudo, Nevadillo negro, Hojiblanca	[45]
Syria	Sorani, Doebli	[46]
Tunisia	Gerboui, Chétoui, Chemlali, Oueslati	[47]
Turkey	Memeli, Çelebi, Gemlik Memecik, Erkence, Çekiste	[48]

**Table 2:** Differences in the chemical composition of *O. europaea* in various plant parts.

Part Used	Plant Extract	Method	Chemical Composition	Reference
Branch	Vegetal extract	HPLC-DAD-MS	Phenolic compounds Oleuropein; comselogoside; flavanonols; taxifolin; hydroxytyrosol; verbascoside; 1-acetoxypinoresinol glucoside; eriodictyol; esculetin Triterpenoids Oleanolic acid, ursolic acid, maslinic acid; and erythrodiol	[50]
Stems	Vegetal extract	RP-HPLC-DAD-ESI-QTOF-MS and -MS/MS	Phenolic compounds Phenolic acids Chlorogenic acid Gallic acid Dihydroxybenzoic acid hexoside Phenolic aldehydes Vanillin Phenylethanoids Hydroxytyrosol Hydroxytyrosol-hexoside II Tyrosol Verbascoside Coumarins Aesculetin Flavonoids Taxifolin Luteolin di-O-hexoside Apigenin 6–8-di-C-glucoside Quercetin Quercetin 3-*O*-rutinoside Luteolin 7-*O*-glucoside Luteolin-7-*O*-rutinoside Chrysoeriol 7-*O*-glucoside Apigenin 7-*O*-rutinoside Chrysoeriol *β*-Hydroxyverbascoside Verbascoside Calceolarioside Iridoids and derivatives Loganic acid glucoside Loganic acid Loganin 7-Deoxyloganic acid Secoiridoids and derivatives Oleuropein Acyclodihydroelenolic acid hexoside Hydroxyoleuropein Oleuropein hexoside Dihydro oleuropein Fraxamoside	[51,52]
Seed	Vegetable oil	Gas Chromatography Bradford Assay Flame photometer LC-HR-MS/MS	Protein Albumin, Globulin, Prolamin and Glutelin Mineral K, Na, P, Mg^2+^ Ca^2+^ Fatty Acid Monounsaturated FA Palmitoleic acid C16:1, oleic acid C18:1, eicosenoic acid C20:1, behenic acid C22:1 Polyunsaturated FA Linolenic acid C18:3, Linoleic acid C18:2 Saturated FA Palmitic acid C16:0, stearic acid C18:0, eicosenoic acid C20:0 and lignoceric acid C24:0 Polar fatty lipid Phospholipid Glycolipid Sphingolipid Acyl sterol glycoside	[8,53]
	Vegetal extract	HPLC-DAD-MS	Phenolic compounds Oleuropein Oleoside 11-methyl ester Ligstroside oleoside Nüzhenide Nüzhenide 11-methyl oleoside	[50,52]
Fruit	Vegetable oil	GC-MS UHPLC-HRMS HPLC-DAD-UV HPLC-MS/MS	Fatty acids Tridecanoic acid Myristic acid Palmitic acid Palmitoleic acid Margaric acid Heptadecenoic acid Stearic acid Oleic acid Linoleic acid Linolenic acid Arachidic acid Eicosenoic acid Tocopherols *δ*-tocopherol *γ*-tocopherol *α*-tocopherol *β*-tocopherol Carotenoids *β*-carotene Volatile compounds Isoprene Pent-1-en-3-one Pentan-3-one (*Z*)-Hex-3-enal (*E*)-Pent-2-enal Hexanal (*E*)-Hex-2-enal *trans*-*β*-Ocimene 3-Ethyloct-1,5-diene *α*-Copaene Phenolic compounds Hydroxytyrosol Tyrosol Vanillic acid Verbascoside Rutin *p*-Coumaric acid Eriodictyol Catechin Naringenin Quercetin Oleuropein Oleocanthalic acid Oleaceinic acid Dadzein Caffeic acid Gallic acid Pinoresinol Luteolin Apigenin	[54,55,56]
	Vegetal extract	HPLC-DAD-UV ICP-MS HPLC-MS/MS	Phenolic compounds Tyrosol Hydroxytyrosol Vanillic acid Gallic acid Caffeic acid Chlorogenic acid Vanillin *p*-coumaric acid Verbascoside Rutin Apigenin-7-*β*-D-glucose Luteolin-7-*β*-D-glucose Luteolin Apigenin Secoiridoids Secologanoside Oleoside-11-methylester Oleuropein aglycone Dihydrooleuropein Oleuropein glucoside 6′-*β*-hexopyranosyloleoside Oleuropein Ligstroside Mineral As, Al, Ba, Fe, K, Mg, Mn, Ti, Zn, Na, Ni, P, Si, Ca, Cu, Cd,	[57,58,59]
Leaves	Essential oil	GC-MS	Monoterpene hydrocarbons *α*-Pinene *α*-Thujene *β*-Thujone Myrcene Oxygenated monoterpenes Linalool Borneol Terpinen-4-ol Hydrocarbons *α*-Cubebene *α*-Copaene Sesquiterpene *β*-Cubebene *β*-Elemene *β*-Caryophyllene (*Z*)-*β*-Farnesene *α*-Humulene Germacrene D *β*-Copaene *β*-Bisabolene *δ*-Cadinene Oxygenated sesquiterpenes Caryophyllene oxide *α*-Cadinol Spathulenol Phenolic compounds Tricosane Tetracosane Hydrocarbons Heptacosane Heneicosane Docosane Pentacosane Eugenol Myristicin Hexacosane Alcohols *n*-Decanol *n*-Dodecanol Acids Hexadecanoic acid Oleic acid Ketones *α*-Ionone (*E*)-*β*-Damascenone *β*-Ionone Esters Linalyl acetate Endo-Fenchyl acetate	[60,61]
	Vegetal extract	HPLC-ESI-TOF and IT/MS LC-MS/MS GC-MS HPLC-DAD-TOF-MS HPLC–TOF-HRMS UHPLC/MS	Phenolic compounds Hydroxytyrosol Tyrosol Tyrosol glucoside Hydroxytyrosol glucoside Verbascoside Phenolic acid 3-Hydroxybenzoic Acid *p*-hydroxybenzoic acid Hydroxyphenylacetic acid 4-Hydroxybenzoic acid Gallic acid Protocatechuic acid Chlorogenic acid Vanillic acid Ferulic acid Salycilic acid Benzoic acid *p*-Coumaric acid Cinnamic acid Syringic acid Gallocatechin Flavonoid Apigenin Catechin Luteolin Rutin Taxifolin Hesperetin Quercetin Diosmetin Aromadendrine Kampferol Eriodictyol Flavan 3-ols Hyperoside Quercetin 7-*O*-glucoside Quercetin 3-*O*-rhamnoside Luteolin-7-*O*-glucoside Luteolin 7-*O*-rutinoside Apigenin-7-*O*-glucoside Apigenin-7-*O*-neohesperidoside Lignans Syringaresinol Pinoresinol Acetoxypinoresinol Triterpenoids Ursolic acid Maslinic acid Oleanolic acid Asiatic acid Corosolic acid Oleanonic acid Secoiridoids and related derivatives Ligstroside Oleuropein Secologanoside Loganoside Elenolic acid Oleacein Methoxyoleuropein Demethyloleuropein Hydroxyoleuropein Oleuropein aglycone Ligstroside aglycone	[10,62,63,64,65,66,67,68,69,70,71,72,73,74,75]

**Table 4:** Antifungal activity of various parts of *O. europaea:* summary of research findings.

Part Used	Extract	Strains	Method	Key Results	Reference
Seed	Vegetal extract	*Candida albicans* *Candida glabrata* *Candida dubliniensis* *Candida parapsilosis* *Candida kreusei* *Aspergillus fumigatus* *Aspergillus niger* *Aspergillus flavus* *Alternaria alternata* *Botrytis cinerea* *Fusarium moniliform* *Mauginiella scaettae* *Magnaporthe grisea* *Penicillium digitatum* *Trichothecium roseum* *Saccharomyces cerevisiae*	Disc diffusion Micro-dilution Trypan blue exclusion method Fluorescent dye exclusion method	Ethyl acetate extract Ø = 10–35 mm Methanolic extract Ø = 9–19 mm	[146]
Fruit	Vegetable oil			MIC = 100–400 µg/mL MFC = 808–1260 µg/mL	[140,147,148]
	Vegetal extract			Aqueous extract Ø = 7–13 mm	[142]
Leaves	Essential oil			ZOI = 7–17 mm MIC = 250–1250 µg/mL	[115]
	Vegetal extract			Aqueous extract Ø = 16–19 mm Ethanolic extract Ø = 12.5–25 mm MIC* = 4687 µg/mL MIC* = 6250 µg/mL	[13,134,135,136,149,150]

Ø = zone of inhibition; MIC = minimum inhibition concentration; MFC = minimum fungicidal concentration; MIC* = concentration yielding 80% growth inhibition compared to the growth in extract-free medium.

**Table 5:** Antioxidant activity of various parts of *O. europaea:* summary of research findings.

Part Used	Extract	Method	Key Results	Reference
Seed	Vegetal extract	Radical scavenging activity 2,2-Diphenyl-1-Picrylhydrazyl (DPPH) 6-sulfonic acid (ABTS) Ferric reducing antioxidant power (FRAP) *β*-Carotene bleaching assay (BCB) H_2_O_2_ scavenging activity Metal ion chelating activity (EDTA) Nitric oxide scavenging activity (NO) Reducing capacity of Fe^3+^/Fe^2+^ conversion	DPPH/IC_50_ = 5.25 µg/mL Fe^3+^/Fe^2+^ IC_50_ = 20.24 µg/mL EDTA/IC_50_ = 17.37 µg/mL H_2_O_2_/IC_50_ = 30.50 µg/mL	[96]
Fruit	Vegetable oil		DPPH = 207.00 mg GAE/kg ABTS = 1.806 ± 0.042 mmol Trolox/kg *β*-Carotene = 65.41% of inhibition	[160,161]
	Vegetal extract		DPPH/IC_50_ = 9.47 µg/mL Fe^3+^/Fe^2+^ IC_50_ = 77.25 µg/mL EDTA/IC_50_ = 24.76 µg/mL H_2_O_2_/IC_50_ = 36.39 µg/mL	[96]
Leaves	Vegetal extract		DPPH/IC_50_ = 0.034–1.38 mg/mL EDTA/IC_50_ = 0.155 mg/mL FRAP/IC_50_ = 0.0753–1.018 mg/mL ABTS/IC_50_ = 0.0187–1.647 mg/mL NO/IC_50_ = 0.15–1.00 mg/mL	[19,119,162,163,164,165,166]

**Table 8:** Cytotoxicity activity of various parts of *O. europaea:* summary of research findings.

Parts Used	Extracts	Cell Line	In Vitro Model	Key Results	Reference
Seed	Methanolic	Human colorectal cell (HCT-116) Human melanoma cells (501 Mel) Colon cancer cell line (HT29) Prostate cancer cell line (PC3) Breast cancer cell line (MCF-7) Malignant mesothelioma cell line (REN) Hepatocellular cancer cell line (HEPG-2) Human promyelocytic leukemia cells (HL-60) Human glioblastoma cell line (T98G) Human lung cancer cell lines (A549) Pancreatic cancer cell line (AsPC-1) Larynx cancer cell line (HEP2) Luminal A breast cancer Cell line (MCF7) Human chronic leukemia cell line (K562)	MTT and MTS assay Cell cycle in-cell ELISA Kit Cell death detection ELISA^+^ The RNeasy mini kit Resazurin assay Agarose gel electrophoresis Assessment of cell growth inhibition WST assay for cell viability	IC_50_ = 875.5 µg/mL	[210]
Fruit	Methanolic			IC_50_ = 154.3 µg/mL	
	Vegetable oil			IC_50_ = 81.9 ± 6.9 mM IC_50_ = 19.1 ± 5.8 mM	[202]
	Ethanolic			IC_50_ = 1000 µg/mL IC_50_ = 600 µg/mL	[83]
Leave	Aqueous			IC_50_ = 203.1 µg/mL IC_50_ = 236.6 µg/mL	[205]
				IC_50_ = 15.5 ± 0.40 µg/mL IC_50_ = 10.2 ± 0.45 µg/mL IC_50_ = 9.9 ± 0.15 µg/mL IC_50_ = 10.4 ± 0.16 µg/mL IC_50_ = 8.6 ± 0.13 µg/mL	[211]
				IC_50_ = 100 µg/mL	[212]
				IC_50_ = 21.91 ± 1.8 μg/mL	[213]
	Ethanolic			IC_50_ = 200 µg/mL	[214]
				IC_50_ = 100 µM IC_50_ = 40.8 μM IC_50_ = 52 µM	[215]
	Methanolic			IC_50_ = 21.5 mg GAE/L	[209]
				EC_50_ = 0.26 mg/mL	[20]

## Data Availability

All data used are cited within the manuscript.
